# Comparison of traditional instruction versus nontraditional learning to improve trainee knowledge of urine culture practices in catheterized patients

**DOI:** 10.1017/ash.2022.225

**Published:** 2022-05-16

**Authors:** Catherine A. Gao, Rupak Datta, Dana Dunne, Louise-Marie Dembry, Richard A. Martinello, Manisha Juthani-Mehta, Sonali D. Advani

**Affiliations:** 1 Division of Pulmonary and Critical Care, Northwestern University Medicine, Chicago, Illinois; 2 Section of Infectious Diseases, Department of Internal Medicine, Yale School of Medicine, New Haven, Connecticut; 3 Department of Infection Prevention, Yale New Haven Health, New Haven, Connecticut; 4 Department of Pediatrics, Yale School of Medicine, New Haven, Connecticut; 5 Veterans’ Affairs Connecticut Healthcare System, West Haven, Connecticut; 6 Department of Epidemiology and Microbial Diseases, Yale School of Public Health, New Haven, Connecticut; 7 Division of Infectious Diseases, Department of Internal Medicine, Duke University School of Medicine, Durham, North Carolina

## Abstract

We surveyed trainees about their urine culture practices and assessed the impact of an educational intervention delivered electronically and in person. Trainee scores improved across all levels of training and across all questions on the postintervention survey, but there was no difference in scores by mode of education (*P* = .91).

The Infectious Diseases Society of America (IDSA) guidelines suggest that most positive urine cultures in catheterized patients represent asymptomatic bacteriuria, which do not require treatment.^1^ Catheter-associated urinary tract infection (CAUTI) is considered a diagnosis of exclusion unless the patient has evidence of genitourinary obstruction or pyelonephritis. In their joint guidance, IDSA and the American College of Critical Care Medicine recommend urine cultures in initial work-up of fever in catheterized patients in high-risk scenarios: (1) genitourinary obstruction, (2) genitourinary surgery, (3) neutropenia, and (4) renal transplantation.^2^ However, there is a large gap between these recommendations and actual clinical practice.^3^ Multifaceted interventions have demonstrated reduction in treatment of asymptomatic bacteriuria (ASB),^4^ but outreach to trainees has been limited. Our objectives were to evaluate current knowledge of trainees related to indications for ordering urine cultures in catheterized patients, and to compare the impact of traditional instruction versus nontraditional modes of learning on their knowledge.

## Methods

### Design

We conducted baseline and posteducation surveys of trainees between January 1, 2018, and March 15, 2018. This study was deemed a quality improvement project by the Yale University Institutional Review Board.

### Setting and respondents

This intervention was conducted at Yale New Haven Hospital, a 1,541-bed, nonprofit, academic, quaternary-care hospital in New Haven, Connecticut. Respondents were trainees including medical students, interns, residents, and fellows.

### Survey instrument and distribution

We adapted a previously validated survey instrument with the assistance of an internal medicine resident (C.A.G.) to assess trainee knowledge about urine culturing in catheterized patient.^5^ The survey (Supplement 1) includes questions related to the respondent’s role: 12 questions related to indications for ordering urine cultures in catheterized patients and 1 question related to panculturing. Response accuracy was assessed using the 2009 IDSA CAUTI and 2005 ASB guidelines as the standard.^6,7^ One question related to new onset confusion was excluded because the 2019 IDSA ASB guidelines update was not published at the time of this survey.^1^

Participation was voluntary and anonymous; survey data analysts did not have any respondent identifiers. The baseline surveys were distributed to 201 trainees starting January 1, 2018, prior to specific conferences by infection prevention staff as described below. The response rate to the baseline survey was calculated using conference attendance as the denominator and number of completed surveys returned as the numerator. Posteducation surveys were distributed in March 2018 electronically to 1,340 trainees through their respective listservs (to capture trainees that did not attend conferences but were exposed to other modes of education). The posteducation survey was the similar to the baseline survey, with the exception of 1 additional question about the mode of education (ie, conference, electronic resources, word of mouth, or none).

### Institutional efforts and education

An educational intervention with an evidence-based urine culture algorithm and the IDSA guidelines were implemented using 2 modalities: (1) traditional in-person instruction through trainee conferences and (2) nontraditional modes of learning such as electronic resources and word-of-mouth communication with cognitive aids (Supplement 2). Traditional education at conferences was done by the Associate Hospital Epidemiologist (S.D.A.), and baseline surveys were completed prior to these conferences. Electronic resources and cognitive aids were distributed by chief residents after the conferences. Word-of-mouth education with cognitive aids were used during pre-rotation orientation or patient-care rounds by unit leadership and infectious disease physicians.

### Analysis

We reported means for continuous variables and percentages for categorical variables, and we compared responses by trainee level and specialty. Comparisons of percent scores and questions between groups were performed using the Student *t* test, analysis of variance (ANOVA), and the χ^2^ test for linear trends, as appropriate. Data were analyzed using GraphPad Prism software (La Jolla, CA).

## Results

We received 168 responses from trainees to our baseline survey, with a response rate of 83.6%. Most responses were from internal medicine and medicine specialties (59.5%): 13% were from surgical specialties; 11% were from pediatrics; 9% were from emergency medicine and anesthesia; and 5% were from neurology trainees. The mean score on the baseline survey for all trainees was 64.7%. Overall scores increased with level of training (Table 1). We received 108 electronic responses from trainees to our posteducation survey. Our baseline and posteducation cohorts had similar demographics with respect to level of training and specialties (Supplement 3).

**Table 1.** Comparison of Baseline and Posteducation Survey Scores

Role	Baseline Survey, %	Posteducation Survey, %	*P* Value
Fellow	67.75	79.17	.15
Resident	67.73	84.33	<.001
Intern	60.32	77.67	<.001
Medical student	45.37	47.44	.82

**Fig. 1.**
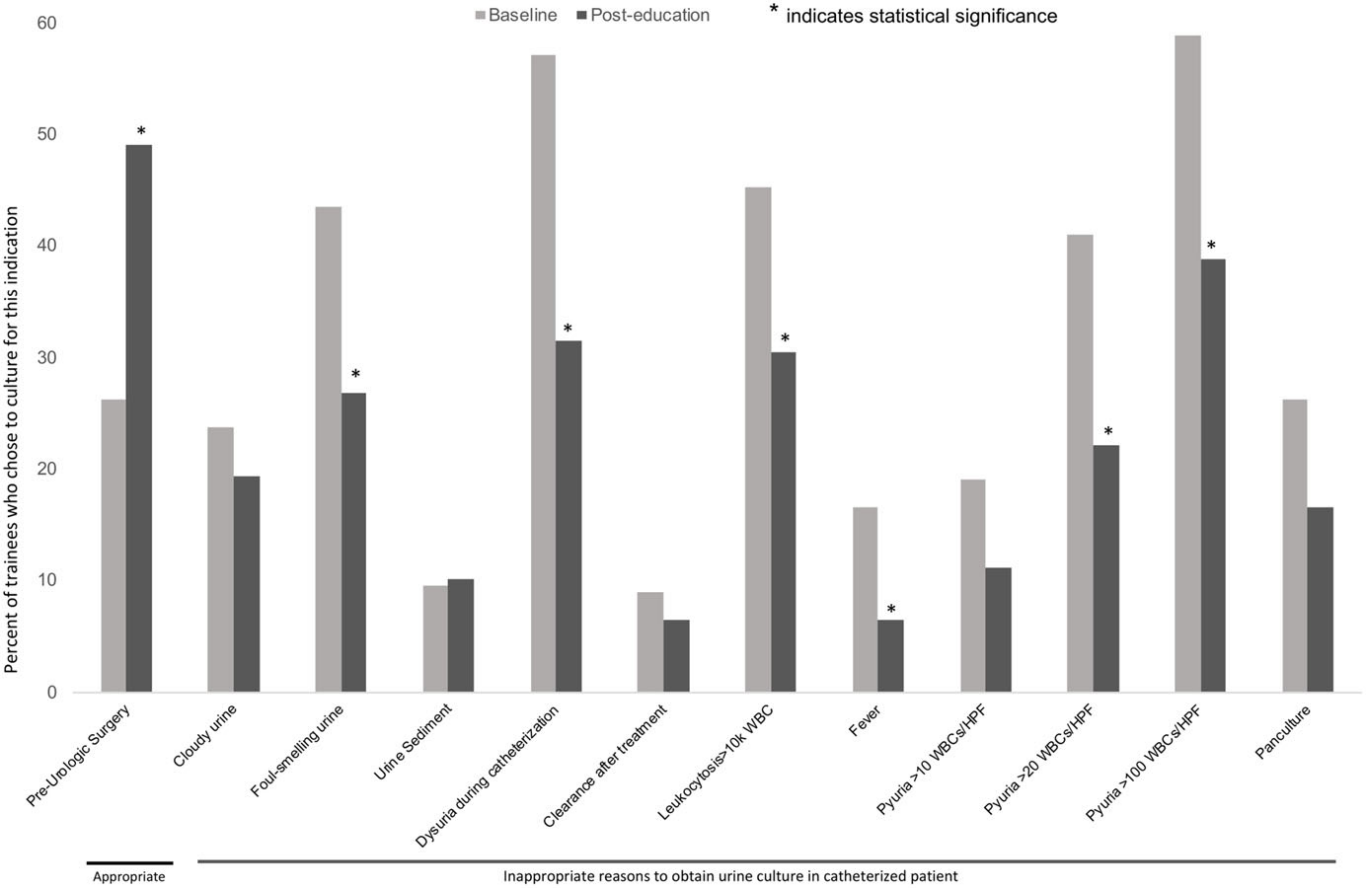
Comparison of baseline and post-education scores by question type.

In the posteducation survey, the overall mean score improved significantly by 12.7% (77.4 vs 64.7%; *P* < .001). Trainee responses to each question also showed improvement (Fig. 1). Residents showed the most improvement in mean scores (16.6% improvement, *P* < .001). However, there was no difference in posteducation scores by mode of education (83.3% conference vs 83.9% word of mouth vs 84.0% electronic resources; *P* = .91). Additionally, trainees who reported exposure to multiple modes of education scored significantly higher (91.7%). However, trainees who did not report exposure to any mode of education scored much lower than others (61.0% vs others; *P* < .01).

## Discussion

Our data reveal gaps in knowledge of evidence-based indications for ordering urine cultures in catheterized patients among medical students, residents, and fellows. This finding is likely due to lack of familiarity with evidence-based guidelines and difficulty in identifying the symptoms of CAUTI. After the educational intervention, scores improved across all levels of training and for all questions; residents showed the most improvement.

Trainee scores on the baseline survey increased in parallel with level of training (67% in advanced trainees). This finding may suggest that trainee knowledge improves with exposure to higher complexity of patients and antimicrobial stewardship. In other data assessing resident knowledge of urine testing, residents similarly scored higher than interns.^8^ These data also highlight the need for incorporating evidence-based guidelines earlier in training and even in medical school. This can be done using a spiral curriculum that introduces the same topics repeatedly, with each encounter increasing in complexity and reinforcing previous learning.^9^

An important finding of the posteducation surveys was that trainee scores improved irrespective of the mode of education. Trainees that received nontraditional forms of instruction (eg, electronic resources) performed similarly to those who attended educational conferences. This finding was unexpected because conferences were more interactive, time-consuming, and allowed for more discussion. Additionally, trainees who reported exposure to multiple modes of education scored significantly higher. These data highlight that e-learning may be a preferred avenue for improving trainee knowledge.^10^ E-learning offers better control over content, sequence, pace of learning, and media, thus tailoring the learner’s experience to match their personal style. Millennial learners also prefer flexible self-directed learning, so electronic resources and cognitive aids may be just as effective as in-person conferences.^11^

This study had several limitations. First, these results from a single academic medical center may have limited generalizability. Secondly, the response rate for our posteducation survey was low, mainly due to use of listservs. However, these responses were complete, and respondent characteristics in our baseline and posteducation survey groups were similar, showing lack of nonresponse bias.^12^ Lastly, the survey responses were deidentified so they could not be individually linked.

In summary, there were significant gaps in knowledge of evidence-based indications for ordering urine cultures in catheterized patients, but trainee knowledge improved with education. Trainees who received nontraditional forms of instruction performed similar to trainees attending traditional conferences. As we integrate stewardship education into medical curricula, nontraditional instruction and multimodal approaches may offer trainees a more individualized experience to suit their learning styles.

## Supplementary material

For supplementary material accompanying this paper visit https://doi.org/10.1017/ash.2022.225. click here to view supplementary material
