# Supplementary material for: Comparison of traditional instruction versus nontraditional learning to improve trainee knowledge of urine culture practices in catheterized patients
Source: Antimicrob Steward Healthc Epidemiol. 2022 May 16;2(1):e81. doi: 10.1017/ash.2022.225 (PMC9139028; doi:10.1017/ash.2022.225)
Supplement: Supplementary file 1 [file ashsup.zip › S2732494X2200225Xsup001.docx]

| Supplement 2: Survey Respondent Demographics for Baseline and Post-education Surveys | | | | | |
| --- | --- | --- | --- | --- | --- |
|  | Baseline Survey  N=168 | | Post-education Survey  N=108 | |  |
| **Role** | N | % | N | % | **P-value** |
| Fellow | 23 | 13.7 | 20 | 18.5 | 0.21 |
| Resident | 94 | 55.9 | 50 | 46.3 | 0.11 |
| Intern | 42 | 25 | 25 | 23.2 | 0.71 |
| Medical Student | 9 | 5.4 | 13 | 12 | 0.04 |
| **Specialty** |  |  |  |  |  |
| Medicine and Medicine Subspecialties | 100 | 59.5 | 53 | 49.1 | 0.10 |
| Surgery and Surgical Subspecialties | 22 | 13.1 | 20 | 18.5 | 0.12 |
| Pediatric specialties | 19 | 11.3 | 14 | 13 | 0.63 |
| Emergency Medicine  and Anesthesia | 15 | 8.9 | 13 | 12 | 0.42 |
| Neurology | 9 | 5.3 | 4 | 3.7 | 0.42 |
| Other | 3 | 1.8 | 4 | 3.7 | 0.39 |
|  |  |  |  |  |  |
