# Supplementary material for: Comparison of traditional instruction versus nontraditional learning to improve trainee knowledge of urine culture practices in catheterized patients
Source: Antimicrob Steward Healthc Epidemiol. 2022 May 16;2(1):e81. doi: 10.1017/ash.2022.225 (PMC9139028; doi:10.1017/ash.2022.225)
Supplement: Supplementary file 1 [file ashsup.zip › S2732494X2200225Xsup002.docx]

**Supplement 1: Thoughtful Urine Cultures in Catheterized Patients**

**(Baseline survey-paper)**

1. Which hospital do you primarily work at?

__________________

1. Are you taking this before or after algorithm rollout/presentation?
2. Before
3. After
4. What is your occupation?
5. Attending
6. Fellow
7. Nurse
8. PA
9. APRN
10. Medical Student
11. Pharmacist
12. Resident PGY1
13. Resident PGY2
14. Resident PGY3
15. Resident PGY4
16. Other (enter comment)
17. What specialty do you primarily work in?
18. Medicine
19. Surgery
20. Trauma
21. Emergency
22. Pediatrics
23. Neurology
24. Anesthesia
25. Other (enter comment)
26. Which units do you work in?
27. Floor
28. ICU
29. Both
30. Other (enter comment)
31. If you work in the ICU, which ICUs do you work in?
32. MICU
33. SICU
34. CCU
35. CTICU
36. NICU
37. Other (enter comment)
38. In which of the following scenarios would you check urine cultures in a CATHETERIZED patient?
39. Cloudy urine
40. Foul-smelling urine
41. Urine sediment in the foley tubing or bag
42. Dysuria while catheterized
43. New confusion in an elderly patient
44. Peri-urologic surgery with anticipated mucosal bleeding such as TURP
45. Follow-up to check for clearance of urinary tract infection
46. Leukocytosis with WBC>10,000 per mm^3^
47. Single temperature to 100.4°F in an immunocompetent patient
48. Pyuria with >10 WBCs/hpf on urinalysis
49. Pyuria with >20 WBCs/hpf on urinalysis
50. Pyuria with >100 WBCs/hpf on urinalysis
51. Other (enter comment)
52. A 50 year old female with recent GI perforation s/p exploratory-laparotomy recovering well in the ICU now has a new fever to 101.4F on post-op day 2. She has a urinary catheter but no signs of obstruction or flank pain. Which approach would you usually take?
53. Pan-culture with blood culture, urine culture, sputum culture, CXR, abdominal imaging
54. Stepwise testing based on history/exam with abdominal imaging and blood cultures first given recent surgery, followed by CXR and sputum cultures if respiratory signs/symptoms, followed by urine cultures if other tests unrevealing
