# Supplementary material for: Comparison of traditional instruction versus nontraditional learning to improve trainee knowledge of urine culture practices in catheterized patients
Source: Antimicrob Steward Healthc Epidemiol. 2022 May 16;2(1):e81. doi: 10.1017/ash.2022.225 (PMC9139028; doi:10.1017/ash.2022.225)
Supplement: Supplementary file 1 [file ashsup.zip › S2732494X2200225Xsup003.pdf]

Urine Culturing Algorithm for Catheterized Patients

Approved by YNHHS System Quality Council on 2/16/2018

Effective:

Yale NewHaven Health

Purpose To provide clinical guidance for assessing patients with indwelling urinary catheters (up to one day after catheter removal) for a urinary tract infection to improve diagnostic accuracy, prevent misdiagnosis and unnecessary exposure to antibiotics and risk of *C.difficile* infections

Background A CAUTI is often a diagnosis of exclusion. The IDSA CAUTI guidelines emphasize the importance of ruling out an alternate cause of a catheterized patient's fever before attributing it to bacteriuria. [1,2]

Patients with indwelling urinary catheters **have up to 10% risk of colonization per day** and frequently show a positive UA (LE, WBC, bacteria, yeast) which does not represent infection. UA should not be used as a guide to order urine cultures in these patients. Repeatedly checking urine cultures in catheterized patients will detect asymptomatic bacteriuria, prematurely narrow the differential diagnosis missing the true infection. [3,4,5]

Are at least one of these signs/symptoms present without other recognizable causes?

- Fever >38.6°C/101.5°F for non-immunocompromised patients
- Fever >38°C/100.4°F for the immunocompromised patient (per CDC definition)
- Altered mental status (after ruling out non-infectious etiology)
- Acute hematuria (after ruling out non-infectious etiology)

NO → **No urine culture or UA with reflex to culture.** Remove indwelling catheter if possible.

YES → **Does the patient meet special criteria?**

- Pregnant/ impending urologic surgery
- Neutropenic, renal transplant
- Costovertebral angle tenderness

YES →

- Remove indwelling catheter
- Consider urine culture (after changing catheter in place for > 7 days)
- Consider blood cultures, respiratory cultures, imaging etc. based on clinical presentation

NO → **Directed evaluation based on signs/symptoms**

- Consider blood cultures, respiratory cultures, imaging etc. based on clinical presentation
- Consider urinalysis-macroscopic w/ reflex to microscopic or simple urinalysis, not UA with reflect to culture or urine culture

→ After first round of directed testing, does patient meet both criteria:

- Persistent fever >38.6°C/101.5°F PLUS
- Source of infection still unclear

NO → **No urine culture or UA with reflex to culture. (Do not use UA as a guide to ordering urine cultures.)** Remove indwelling catheter if possible.

YES →

- Consider urine culture (after changing catheter in place for > 7 days)
- Do not repeat UA or urine culture if ordered once unless significant change in clinical condition
- (Do not order for checking for clearance or test of cure)

Inappropriate urine culture or reflex UA use [7]

- Urine quality: smell, color, sediment
- Elderly patients without fever or urinary symptoms
- To document clearance after treatment
- "Dirty UA", pyuria, bacteriuria
- Leukocytosis or "pan culturing"
- Screening pre or post-operatively (except urologic surgery)

Appendix:

Immunocompromised hosts: primary (congenital) immune deficiencies; uncontrolled HIV infection, cancers associated with immune deficiency, cancer chemotherapy, stem cell or solid organ transplant (SOT), sickle cell diseases, and surgical asplenia; and patients with chronic inflammatory diseases treated with systemic corticosteroid therapy, immunomodulatory medications, and/or biologic agents, neonates and infants less than 3 months of age.

References:

1. Livorsi DJ, Perencevich EN. CAUTI Surveillance: Opportunity or Opportunity Cost? *Infect Control Hosp Epidemiol*. 2015;36(11):1335-1336. doi:10.1017/ice.2015.174
2. Thomas M. Hooton et al, Prevention, and Treatment of Catheter-Associated Urinary Tract Infection in Adults: 2009 International Clinical Practice Guidelines from the Infectious Diseases Society of America, Clinical Infectious Diseases, Volume 50, Issue 5, 1 March 2010, Pages 625–663, https://doi.org/10.1086/650482
3. Eddland A, Hedelin H. Bacterial colonization of the lower urinary tract in women with long-term indwelling urethral catheter *Scand J Infect. Dis.* 1983;15(4):361-365.
4. Warren JW, Tenney JH, Hoopes JM, Muncie HL, Anthony WC. A prospective microbiologic study of bacteriuria in patient with chronic indwelling urethral catheters. *J Infect Dis.*1982;146(6):719-723.
5. Mullin KM, et al. A Multifaceted Approach to Reduction of Catheter-Associated Urinary Tract Infections in the Intensive Care Unit with an Emphasis on "Stewardship of Culturing". *Infect Control Hosp Epidemiol.* 2017;38(2):186-188.
6. Averich et al. Cather-Associated Urinary Tract Infections: Definitions and Significance in the Urologic Patient American Urological Association White Paper
7. Fakih MG, Khatib R. Improving the Culture of Culturing: Critical Asset to Antimicrobial Stewardship, *Infect Control Hosp Epidemiol.* 2017;38(3):377-379.

This is a clinical practice guideline developed by YNHHS CAUTI Committee and helps guide but not replace provider assessment, and excludes sepsis BPA alert.
